# Influence of Pharyngeal Anaesthesia on Post-Bronchoscopic Coughing: A Prospective, Single Blinded, Multicentre Trial

**DOI:** 10.3390/jcm10204773

**Published:** 2021-10-18

**Authors:** Maik Häntschel, Mariella Zahn-Paulsen, Ahmed Ehab, Michael Böckeler, Werner Spengler, Richard A. Lewis, Hubert Hautmann, Jürgen Hetzel

**Affiliations:** 1 Department of Medical Oncology and Pneumology, Eberhard Karls University, D-72076 Tübingen, Germany; mariella.zapa@web.de (M.Z.-P.); dr.a.ehab@gmail.com (A.E.); m.boeckeler@medius-kliniken.de (M.B.); werner.spengerl@med.uni-tuebingen.de (W.S.); juergen.hetzel@med.uni-tuebingen.de (J.H.); 2 Department of Internal Medicine—Pneumology, Cantonal Hospital Winterthur, CH-8401 Winterthur, Switzerland; 3 Department of Anesthesiology and Critical Care, Hospital of Karlsruhe, D-76133 Karlsruhe, Germany; 4 Department of Pneumology, Klinik Loewenstein, D-74245 Loewenstein, Germany; 5 Chest Medicine Department, Mansoura University, Mansoura 35516, Egypt; 6 Department of Internal Medicine, Gastroenterology and Tumor Medicine, Medius Hospitals, D-73760 Ostfildern-Ruit, Germany; 7 University of Worcester, Worcester WR2 6AJ, UK; lewisr@doctors.org.uk; 8 Clinic for Internal Medicine and Pneumology, Hospital Ottobeuren, D-87724 Ottobeuren, Germany; hautmann@email.de

**Keywords:** bronchoscopy, pharyngeal anaesthesia, cough

## Abstract

Background: Local anaesthesia of the pharynx (LAP) was introduced in the era of rigid bronchoscopy (which was initially a conscious procedure under local anaesthetic), and continued into the era of flexible bronchoscopy (FB) in order to facilitate introduction of the FB. LAP reduces cough and gagging reflex, but its post-procedural effect is unclear. This prospective multicentre trial evaluated the effect of LAP on coughing intensity/time and patient comfort after FB, and the feasibility of FB under propofol sedation alone, without LAP. Material and methods: FB was performed in 74 consecutive patients under sedation with propofol, either alone (35 patients, 47.3%) or with additional LAP (39 patients, 52.7%). A primary endpoint of post-procedural coughing duration in the first 10 min after awakening was evaluated. A secondary endpoint was the cough frequency, quality and development of coughing in the same period during the 10 min post-procedure. Finally, the ease of undertaking the FB and the patient’s tolerance and safety were evaluated from the point of view of the investigator, the assistant technician and the patient. Results: We observed a trend to a shorter cumulative coughing time of 48.6 s in the group without LAP compared to 82.8 s in the group receiving LAP within the first 10 min after the procedure, although this difference was not significant (*p* = 0.24). There was no significant difference in the cough frequency, quality, peri-procedural complication rate, nor patient tolerance or safety. FB, including any additional procedure, could be performed equally well with or without LAP in both groups. Conclusions: Our study suggests that undertaking FB under deep sedation without LAP does to affect post-procedural cough duration and frequency. However, further prospective randomised controlled trials are needed to further support this finding.

## 1. Introduction

Flexible bronchoscopy (FB) has been the standard procedure for diagnosis and therapy of a wide variety of respiratory diseases for over 50 years [1]. The use of local anaesthesia (LA) allowed FB to be undertaken without general anaesthesia or sedation by suppressing the cough and gag reflex. However, midazolam, propofol or both have been increasingly used for conscious sedation FB, with or without opiate analgesia, which has improved tolerance, comfort and ease of FB. Nevertheless, local anaesthetics such as lidocaine or oxybuprocaine are still recommended as standard of care [2,3,4,5]. Local anaesthesia is applied to the pharynx (LAP), the larynx, the trachea and endobronchially in order to reduce local mucosal irritation and coughing, which can be most distressing for patients [6]. However, it is unclear whether there is any additional benefit of LAP when other potent forms of sedation are being used. Furthermore, there are no substantial data to indicate that the use of LAP actually influences post-interventional cough.

While side effects of LA are rare [7], there have been reports of serious and life threatening side effects including cardiac [8,9], pulmonary [10] and central nervous [11,12,13,14] impairment, methemoglobinemia [15,16] and allergic reactions [17,18,19,20,21,22,23]. Data on the risk and benefit of LA in addition to general sedation for FB is limited and inconclusive, ranging from reduced cough rate with lidocaine [24] and improved patient tolerance and satisfaction of the procedure [25,26], to a lack of any difference between LA and placebo for bronchoscopy in patients under deep sedation [27]. The British Thoracic Society guideline recommends the use of lidocaine in a concentration of 1% [4] while the American College of Chest Physicians consensus statement endorses the usage of lidocaine in a concentration of 1–10% as effective [28].

This prospective, multicentre, single blinded study was designed to evaluate whether LAP was of any additional benefit when undertaking bronchoscopy under deep sedation, focusing on post-procedural coughing as an indication of patient’s distress.

## 2. Methods

### 2.1. Study Design and Population

The study was undertaken in the pulmonary divisions at the University Hospital Tuebingen, Germany, and the Technical University of Munich, Germany. The study was approved by the staff council and data protection office at the University Hospital Tuebingen, as well as being categorised as a quality assurance evaluation by the ethics committee of the University of Tuebingen, since both bronchoscopic procedures—with and without LAP—represent established standard bronchoscopic practice. 

Patients with an indication for diagnostic or therapeutic bronchoscopy who met inclusion and exclusion criteria were included in the study. Inclusion criteria were the indication for diagnostic or therapeutic bronchoscopy as defined by the local pulmonologist, prior written informed consent, and age of 18 years or above. Exclusion criteria were severe cardiac (e.g., cardiac insufficiency NYHA III-IV, instable angina pectoris), hepatic or psychiatric disorders, myasthenia gravis, pregnancy or nursing period, or known hypersensitivity, allergy or other contraindications for the application of propofol, xylocaine or oxybuprocaine, or its preserving agents. Application of antitussive drugs was not allowed.

To date, it is unclear which values of duration, frequency, type and their distribution can be expected for post-bronchoscopic cough. For sample size calculation, we referred to previous data of Antoniades et al. [24], which examined a comparable setting. Based on this, we aimed for a cohort of 35 individual patients.

### 2.2. Intervention/Procedure

Bronchoscopy was performed using flexible scopes. All patients were intubated with a flexible endotracheal tube (Rüsch BronchoFlex ID 7.5/8.5 mm, Kernen, Germany) to secure the airway and increase patient safety, and to allow bronchoscopic interventions such as cryobiopsies. Two standard procedures were defined:

S1 Flexible bronchoscopy via flexible endotracheal tube with LAP
S2 Flexible bronchoscopy via flexible endotracheal tube without LAP

Both the S1 and S2 regimes were used in blocks of a minimum of 10 consecutive patients, although the order was not randomised. According to the predefined standard (S1 or S2), the allocation of patients was conducted starting with different standards in both centres. The standard was changed only once in each centre (compare Appendix A, Table A1).

All patients were blinded for the usage of LAP, as were the assistant technicians during post-bronchoscopic monitoring, although the bronchoscopist was aware whether or not the patient had received LAP. Propofol was used in all patients, as with an initial bolus of approximately 1 mg propofol per kg of body weight, followed by a continuous propofol infusion with a further propofol boli if needed. After initiation of propofol, either procedure S1 or S2 was applied. 

S1: The bronchoscope was inserted perorally into the patient’s pharynx. 10 mL of 1% oxybuprocaine was applied via the bronchoscope’s working channel to the pharynx and the larynx, including the vocal cords, and the bronchoscope was removed completely. One minute later, the bronchoscope was reinserted, the patient was intubated by sleeving the endotracheal tube over the bronchoscope and local anaesthesia was instillated endobronchially.

S2: The bronchoscope was inserted perorally into the patient’s pharynx. Without application of any LAP, the patient was intubated by sleeving the endotracheal tube over the bronchoscope and local anaesthesia was instillated endobronchially.

The subsequent bronchoscopy proceeded in the usual fashion in both S1 and S2 arms of the study. All bronchoscopies were performed by the experienced investigators M.B., W.S. and J.H. in Tuebingen and H.H. in Munich. All patients were intubated with a 7.5 mm or 8.5 mm oropharyngeal tube. Supplemental oxygen was given by a nasal cannula and adjusted in order to maintain an oxygen saturation (SpO_2_) of 95% or above. Blood pressure was measured non-invasively, monitored at 3 min intervals until the patient was extubated and transferred in the recovery room.

### 2.3. Cough Monitoring

Immediately after arrival in the recovery room, an audio recording was started with a microphone placed at a distance of 10–20 cm from the patient’s mouth (Sony ICD-PX240). The duration of recording was 10 min. Coughing was evaluated for duration and characterisation by M.H. and M.Z.-P. using Sony Sound Organizer Version 1.5.0.10210 software in a blinded fashion.

**Figure A1:**
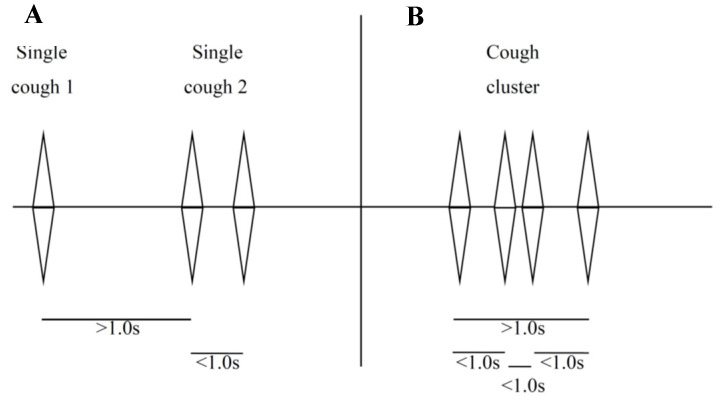
(**A**)—Example for single coughs. One cough or multiple coughs with a total duration < 1.0 s; (**B**)—Example for cough clusters. It shows numerous subsequent forced exhalations (interval between each subsequent forced exhalations of less than one second, but with a total duration of more than one second). A cough cluster starts with the first forced exhalations and ending with the last forced exhalation.

Cough intensity was quantified by the cumulative duration of separate coughs over the 10 min period. A single cough was defined as one cough or multiple coughs with a total duration less than 1.0 s. A cough cluster was defined as numerous subsequent forced exhalations with an interval between each subsequent forced exhalation of less than one second, but with a total duration of more than one second (Appendix A, Figure A1).

### 2.4. Data Acquisition

Inter- and post-procedural monitoring followed the local standard, which included duration of procedure, initial and lowest blood pressure, initial and lowest SpO_2_ and mean oxygen supplementation [litres per minute], both during and after bronchoscopy. Propofol dose was documented.

### 2.5. Evaluation of Patient’s Tolerance and Comfort

Feasibility of procedure, tolerance of bronchoscopy and subjective assessment of post-procedural coughing and gagging were evaluated with questionnaires for the bronchoscopist, the assistant technician and the patient. Answers were given on a 5-step visual analogue scale for physicians and assistant technicians and an 11-step visual analogue scale for the patients, ranging from “not at all” to “most severe” (e.g., cough, gag).

### 2.6. Endpoints

Coughing duration during the first 10 min in the monitoring room after the procedure was defined as primary endpoint. Secondary endpoints were number of coughs and cough clusters, distribution of single coughs and cough clusters and development of coughing during the 10 min post-procedure based on the audio recording.

### 2.7. Statistical Analysis

Differences between both standards for cumulative duration of coughing, cough characteristics, patient’s tolerance and comfort were calculated by the Wilcoxon Test/Chi-square test using JMP 14.2.0 software. Variate analysis was calculated by Kendall’s rank correlation coefficient test. A *p*-value < 0.05 was considered as significant.

## 3. Results

### 3.1. Study Population

**Figure A2:**
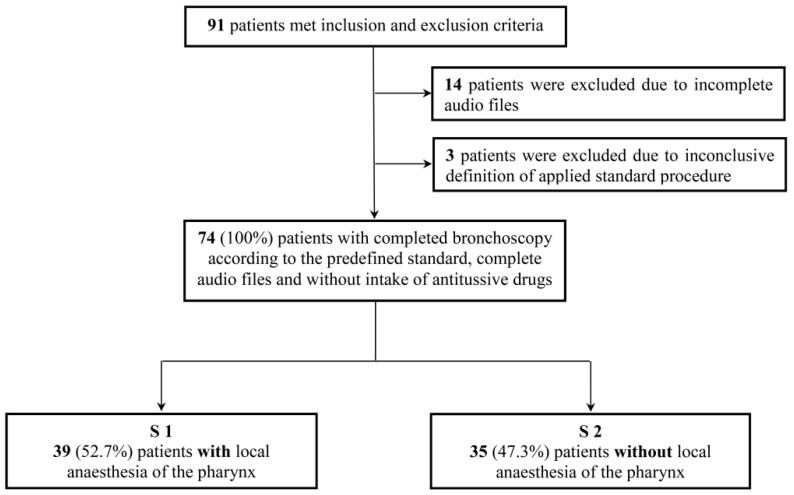
Study population. S1—Standard 1 with LAP, S2—Standard 2 without LAP.

The composition of the study population is shown in Appendix A, Figure A2. A total of five patients were examined with the standard other than the predefined standard—three patients with LAP at predefined standard S2, two patients without LAP at predefined standard S1 (Appendix A Table A1).

### 3.2. Patient Characteristics

The baseline characteristics for both groups of patients are shown in Table 1. There were no significant differences between the groups for mean age, body mass index, smoking history (comparing never smoker with ever smoker) and pre-existing cardiac or pulmonary diseases. The number of male patients was significantly higher in group S2 compared to group S1.

### 3.3. Bronchoscopic Procedure

The bronchoscopic interventions, sedation regimens, duration of procedure and peri-procedural and post-procedural monitoring are summarised in Appendix A, Table A2. In most cases, two or more interventions were performed. The mean duration of bronchoscopy did not significantly differ between either group. There was no difference either in absolute amount or in the propofol application per minute. Peri- and post-procedural monitoring and mean oxygen supplementation did not significantly differ between both groups.

### 3.4. Cough Duration and Development

**Figure 1:**
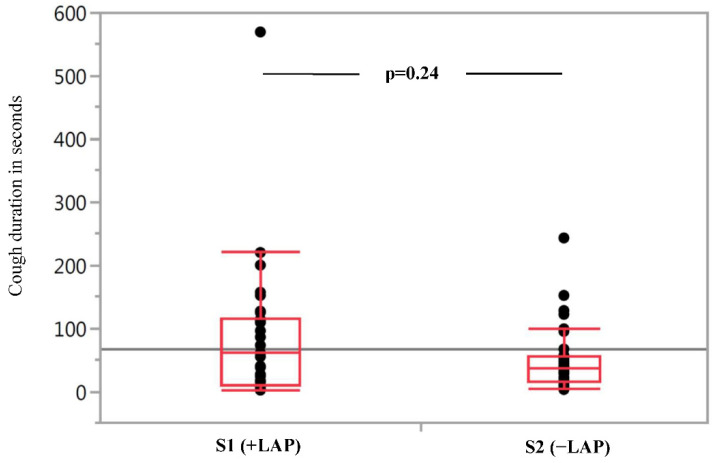
Cumulative cough duration in seconds during the observation interval of 10 min immediately after extubation. Each dot represents the cough duration of a single patient in seconds. S1 (+LAP)—Standard 1 with local anaesthesia of the pharynx, S2 (−LAP)—Standard 2 without local anaesthesia of the pharynx.

The cumulative cough duration varied widely between patients in each group, with no normal distribution. Figure 1. shows the median cumulative cough duration in seconds during the ten minute period immediately after extubation as primary endpoint of the study. The duration of coughing in S1 (with LAP) was 61 s. The median cumulative cough duration in S2 (without LAP) was shorter at 38 s. However, the difference between S1 and S2 did not reach a significant level (*p* = 0.24).

**Figure 2:**
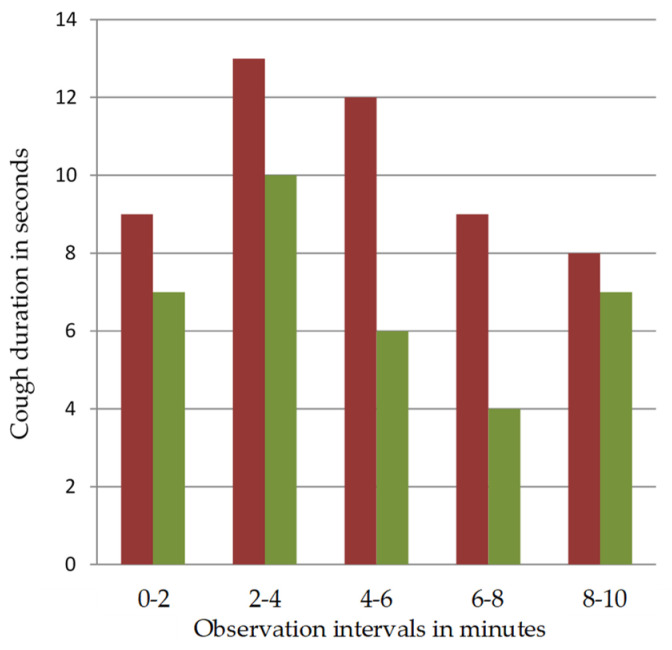
Development of median cumulative cough duration in 2-min intervals during the 10 min recording time. Median cumulative cough duration is shown in seconds for both Standards (S1 (+LAP)—red columns, S2 (−LAP)—green columns) for the respective intervals.

We categorised cough duration in five 2-min intervals for the entire 10 min recording time to evaluate development of coughing over time. Median cumulative cough duration showed a shorter duration in S2 for each single 2-min interval compared to S1, but again, did not reach a statistically significant level (Figure 2).

### 3.5. Single Coughs and Cough Clusters

**Figure A3:**
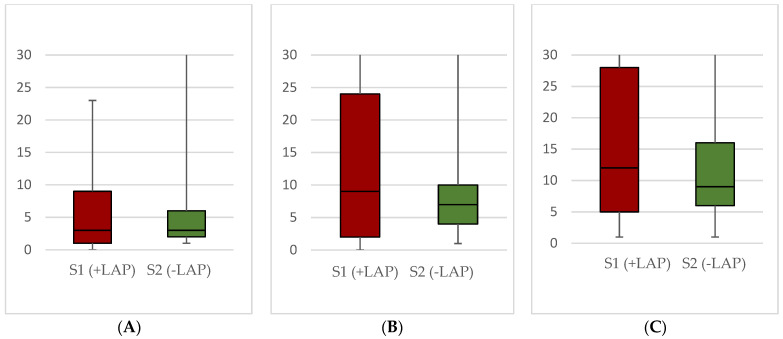
Box plots show median and quantiles for absolute number of single coughs (**A**), cough clusters (**B**) and the sum of both (**C**) for each patient during the 10-min evaluation interval, separately for both standards (S1 (+LAP)—Standard 1 with local anaesthesia of the pharynx; S2 (−LAP)—Standard 2 without local anaesthesia of the pharynx).

As coughing is not uniform and may vary both inter- and intra-individually, we categorised coughing in single coughs and cough clusters (Appendix A, Figure A1) to analyse different cough characteristics. Appendix A, Figure A3 shows the cumulative number of single coughs (A), cumulative number of cough clusters (B) and the sum of both during the 10 min recording period. Median absolute number of single coughs during the 10 min was the same for both groups (*n* = 3). The median of cough clusters (+LAP: *n* = 9; −LAP: *n* = 7) was lower for patients without LAP but did not reach a significant level. The distribution range of the middle quantiles showed a larger spread in the cohort with LAP compared to the cohort without LAP.

### 3.6. Patient’s Tolerance, Comfort and Complaints

**Figure A4:**
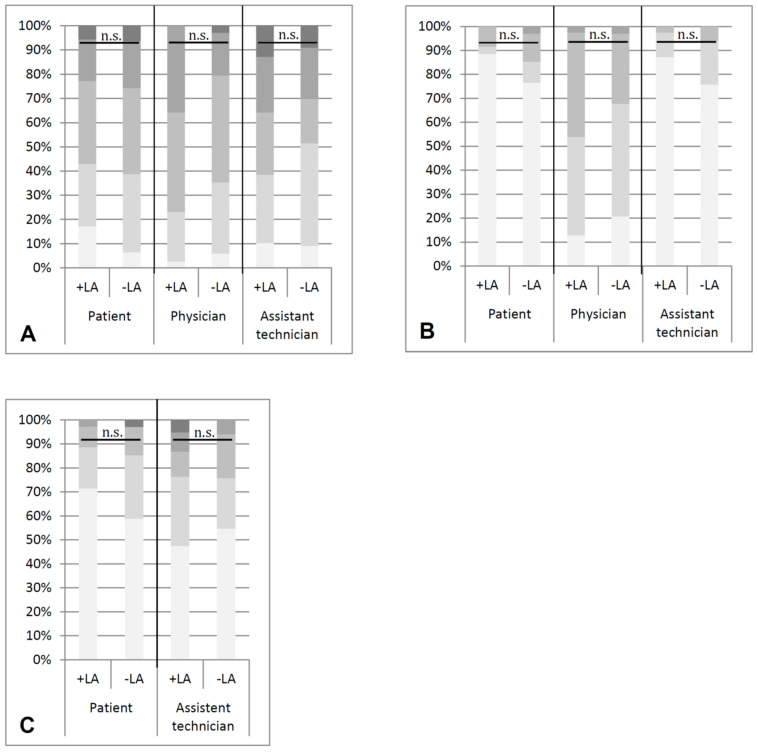
(**A**–**C**) Postinterventional complaints—coughing (**A**), gagging (**B**) and dyspnea (**C**). (**A**) Assessment of patient’s coughing, semi-quantified on a 5-step scale by the patient himself, the investigator (doctor) and the assistant technician in the recovery room. The scale ranged from 0 (no—light colour) to 4 (maximum—dark colour) for S1 (+LAP) as red columns and for S2 (−LAP) as green columns. (**B**) Assessment of patient’s gagging, semi-quantified on a 5-step scale by the patient himself, the investigator (doctor) and the assistant technician in the recovery room. The scale ranged from 0 (no—light colour) to 4 (maximum—dark colour) for S1 (+LAP) as red columns and for S2 (−LAP) as green columns. (**C**) Assessment of patient’s dyspnea, semi-quantified on a 5-step scale by the patient himself and the assistant technician in the recovery room. The scale ranged from 0 (no—light colour) to 4 (maximum—dark colour) for S1 (+LAP) as red columns and for S2 (−LAP) as green columns. n.s.—not significant.

Appendix A Figure A4 shows post-procedural complaints (dyspnea, gagging and coughing) for both standard procedures as assessed by the patient, assistant technician and physician. Due to the infrequency of moderate or severe complaints, patient scores were grouped into five (very mild (0–1); mild (2–3); moderate (4–6); severe (7–8) and very severe (9–10)). We observed no significant difference for coughing and gagging or dyspnea between either group nor when assessed by the patient investigator or assistant technician.

### 3.7. Coughing by Age

**Figure 3:**
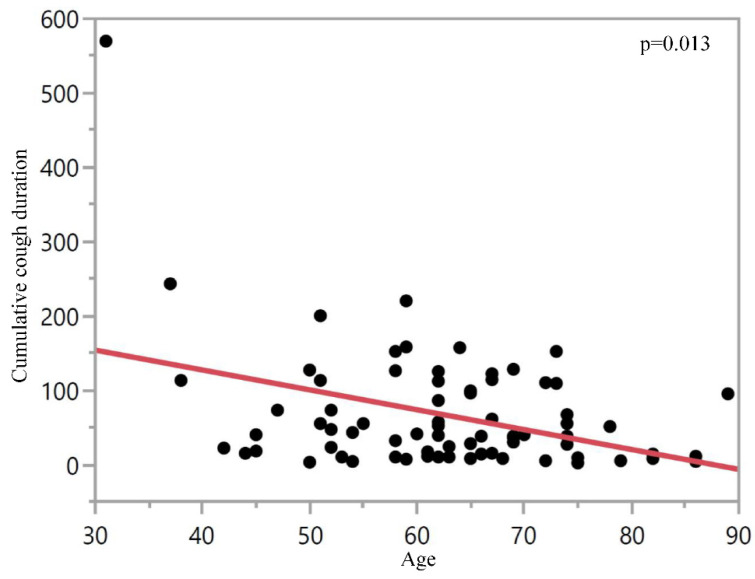
Cumulative cough duration in seconds during the ten minute post-procedural observation period with regard to patient age. Shown data cumulate both groups (S1 and S2, independent of application of LAP) with one dot for each patient.

The multivariate analysis for cough duration as primary endpoint (taking into consideration baseline characteristics, procedures, sedation and peri-procedural monitoring for the whole study population—irrespective of the application of LAP) revealed age as an independent variable, associated with reduced cough duration (*p* = 0.013) (Figure 3).

### 3.8. Adverse Events

Adverse events were evaluated during the intervention as well as post-procedurally in the recovery room. Besides two episodes of desaturation during bronchoscopy without LAP, which could be stabilised by increasing the oxygen flow, neither serious adverse events nor any other adverse events occurred during the bronchoscopy in either group (Appendix A Table A3).

During the post-procedural surveillance, we observed ten adverse events (29.4%) in S1 with LAP and five adverse events (16.7%) in the S2 without LAP. All adverse events in both cohorts could be related to the bronchoscopic intervention as anticipated events and were easily managed. No adverse event was associated with the application of LAP. There were no post-procedural serious adverse events in either group. There were no longer lasting health problems associated with the procedure.

## 4. Discussion

This prospective multicentre study demonstrates that bronchoscopy under deep sedation with propofol is feasible without additional application of LAP. Although there was a trend for reduced post-procedural coughing without LAP, this was not significant. Since there were no reported safety concerns, and because patient tolerance was the same with or without LAP, we suggest that LAP may not be needed, and indeed may be disadvantageous due to side effects when a patient is under deep sedation during the bronchoscopic procedure.

Application of LAP has been used in flexible bronchoscopy for decades and is considered to be standard practice in order to improve patient tolerance and comfort. However, its side effects, albeit rare, may sometimes be serious and are not usually considered [7,8,9,10,11,12,13,14,15,17,18,19,20,21,22,23]. Although the application of LA has been recommended in various guidelines, data supporting its advantage are limited in the context of deep sedation [4,29]. While LAP is mandatory in flexible bronchoscopy without sedation, it has been unclear whether this should remain true when bronchoscopy is performed under deep sedation.

There is only one study that addressed the use of LA in patients under sedation during bronchoscopy. In this small, randomised trial with 49 patients, Antoniades et al. found a reduced cough rate per minute and a reduced dose of sedating drugs when using topical lidocaine compared to placebo [24]. However, cough rate was determined during the bronchoscopy and benzodiazepines were used for sedation at low total dose (mean midazolam 3.4 mg and 2.1 mg, respectively), thus producing only low sedation depth. This study is not comparable with the current procedure with preferential use of propofol for sedation during bronchoscopy.

Using blinded evaluation in our study, we were able to characterise post-procedural cough in more detail for the first time in terms of its duration, frequency and presentation.

The trend towards less coughing after bronchoscopy in the group without LAP may be explained by the lack of local anaesthesia in the pharynx, since local anaesthesia in the pharynx may result in a reduced or completely suppressed swallowing reflex. Therefore, after bronchoscopy, when the patient is coughing up secretions, it is possible that those patients who have suppressed swallowing reflex and residual anaesthesia of the vocal cords may be more prone to aspiration with further need to clear the large airway. This hypothesis is supported by the fact that there is a trend towards more coughing clusters in the group with LAP, whereas single coughs did not at all differ between both groups (Appendix A, Figure A3).

There were no baseline characteristics or differences in bronchoscopic procedures which could have affected the results (Table 1, Appendix A, Table A2).

It is of note that the lack of LAP did not impair the bronchoscopy procedure and all procedures were performed as planned. There was no difference in patient tolerance, cough intensity or gagging during bronchoscopy. Moreover, the subjective tolerance of the patients, which is considered as one of the key points, did not differ between the groups. Since these subjective assessments were consistent in all three assessment groups, this supports the validity of the results.

There were more adverse events (*n* = 10 (29.4%)) after bronchoscopy in the group with LAP, although it is not possible to be sure whether these were in any way related to the use of LAP due to relatively small numbers.

An additional advantage of deep sedation is that it allows orotracheal intubation via the bronchoscope, which was performed in almost all patients to increase overall safety and efficacy of the procedure. Furthermore, orotracheal intubation enables large volume material to be removed from the airway, which would otherwise not be possible via the working channel of the bronchoscope, in addition to other more complex bronchoscopic interventions such as endobronchial recanalisation, stenting and transbronchial cryobiopsy. In less deeply sedated patients, LAP may be of greater relevance, therefore these results may not be transferable to mildly sedated patients without intubation.

Based on our multivariate analysis, we identified age as a single variable which is associated with less coughing (Figure 3). To our knowledge, this has not been reported in a prospective study setting so far. One explanation is that reduced pharyngeal and supraglottic sensitivity with increasing age may be associated with increased aspiration [30,31]. Application of LAP in elderly patients needs to be considered carefully as the combination of LAP-induced mucosal hyposensitivity with pre-existing diminished local sensitivity [32] may increase the risk of aspiration and retention of endobronchial substances.

Our study has a number of limitations that have to be considered. Firstly, because this was a real-life study, only patients and assistant technician staff in the recovery room were blinded for the application of LAP, but not the physicians during bronchoscopy. Secondly, although patients were allocated to the predefined standard procedure over predefined time periods, there was no randomisation. This may have led to a relevant selection bias. Additionally, five patients were treated by other than the initially intended cohort allocation (Appendix A Table A1). However, we want to emphasise that not all five patients changed the group in the same direction; three patients got LAP who were allocated to S2, and two patients did not receive LAP who were allocated to S1. Since the size of the tubes was not recorded, an unequal distribution between the groups cannot be ruled out which may have affected the result. Finally, low case numbers are a limitation.

## 5. Conclusions

We conclude there is no need to use LAP for patients during flexible bronchoscopy when the procedure is performed under deep sedation. The addition of LAP may result in more side effects and more coughing during the post-bronchoscopic period.

## Figures and Tables

**Table 1:** Baseline characteristics for the two groups—S1 (Standard 1 with LAP) versus S2 (Standard 2, without LAP).

Baseline Characteristics *			
	S1 (with LAP)	S2 (without LAP)	*p*-Value
Variable	*N* = 39 (100%)	*N* = 35 (100%)	
Age—years	61.6 ± 11.4	63.7 ± 12.2	n.s.
Sex—no. (%) ^			
Male	13 (46.4)	20 (76.9)	0.02
Female	15 (53.6)	6 (23.1)	
Body weight—kg	71.5 ± 17.9	74.1 ± 12.7	n.s.
Body height—cm	169.5 ± 8.6	171.7 ± 9.5	n.s.
Body mass index—kg/m^2^	24.8 ± 5.5	25.2 ± 4.1	n.s.
Smoking status—no. (%)			
Never smoker	12 (30.8)	7 (20.0)	0.36
Ever smoker	27 (69.2)	28 (80.0)	
Former smoker	13 (33.3)	17 (40.0)	
Current Smoker	14 (35.9)	11 (40.0)	
Preexisting lung disease—no. (%)			
Yes	31 (79.5)	22 (62.9)	0.11
No	8 (20.5)	13 (37.1)	
Preexisting cardiac disease—no. (%) ^			
Yes	8 (21.1)	10 (29.4)	0.41
No	30 (78.9)	24 (70.6)	

* age, body weight, body height, body mass index and smoking history are presented as mean ± standard deviation (SD); sex, smoking status, pre-existing lung disease, pre-existing cardiac disease and performance status are presented as absolute number and percentage (%). ^ Baseline characteristics were unknown for sex in 11 patients in S1 and 9 patients in S2; pre-existing cardiac disease was unknown in S1 in one case, performance status was unknown in S2 in one case. LAP—local anaesthesia of the pharynx.

## Data Availability

The data presented in this study are available on request from the corresponding author.

## Appendix A

**Table A1:** Sequence of patient assignment. S1—Standard 1 with LAP, S2—Standard 2 without LAP; LAP—local anaesthesia of the pharynx.

Sequence of Patient Assignment for Each Centre			
Centre	Predefined Standard	Patients Received …	
		S1 (+LAP)	S2 (−LAP)
1	S1	25	1
	S2	3	25
2	S2	0	8
	S1	11	1
		39	35

**Table A2:** Bronchoscopic interventions including procedures, medication and peri-interventional monitoring for the two groups—S1 (Standard 1 with LAP) versus S2 (Standard 2, without LAP).

Procedures, Sedation and Peri-interventional Monitoring *				
		S1 (with LAP)	S2 (without LAP)	*p*-Value
		*N* = 39 (100%)	*N* = 35 (100%)	
Procedures—no. (%)		38 ^1^	34 ^1^	n.s.
BL or BAL without additional procedure		12 (31.6)	12 (35.3)	
BL or BAL + additional procedure		19 (50.0)	15 (44.1)	
EBB/TBB		13 (34.2)	10 (29.4)	
TBNA		1 (2.6)	3 (8.8)	
EBB/TBB + TBNA		5 (13.2)	2 (5.9)	
Others °		7 (18.4)	7 (20.6)	
**Sedation with propofol—no. (%)**		39 (100)	34 (100) ^1^	
Absolute dose of propofol—mg		242.7 ± 140	261.5 ± 108.8 ^1^	n.s.
Propofol dose/time—mg/min		17.0 ± 9.7 ^1^	17.4 ± 10.0 ^7^	n.s.
**Intubation yes—no. (%)**		36 (100) ^3^	31 (96.9) ^3^	n.s.
**Duration of intervention—min**		17.9 ± 10.7 ^1^	19.2 ± 11.7 ^6^	n.s.
**Peri-interventional monitoring**				
MAP—mmHg	pre-interventional	98.5 ± 2.3	102.4 ± 2.5	n.s.
	lowest	79.8 ± 14.0 ^4^	85.5 ± 16.1 ^5^	n.s.
SpO_2_—%	pre-interventional	94.8 ± 4.0 ^1^	95.0 ± 4.2	n.s.
	lowest	89.4 ± 6.1	86.0 ± 11.5 ^4^	n.s.
Oxygen supply—l/min		5.0 ± 1.3 ^1^	5.3 ± 2.2 ^4^	n.s.
**Postinterventional monitoring**				
MAP—mmHg	initial	87.2 ± 2.7	85.0 ± 2.9 ^1^	n.s.
	lowest	78.6 ± 13.7 ^3^	83.3 ± 14.2 ^14^	n.s.
SpO_2_—%	initial	93.3 ± 6.1 ^1^	93.2 ± 4.9 ^1^	n.s.
	lowest	91.1 ± 0.9 ^2^	92.0 ± 1.1 ^8^	n.s.

* Data for sedation, peri-interventional monitoring and post-interventional monitoring are presented as mean ± standard deviation (SD); procedures are presented as absolute number and percentage (%). BAL—bronchoalveolar lavage; BL—bronchial lavage; EBB—endobronchial biopsy; MAP—mean arterial pressure; SpO_2_—oxygen saturation; TBB—transbronchial biopsy; TBNA—transbronchial needle aspiration; n.s.—not significant. ° Other interventions for S1 include one patient with removal of valves, one patient with bronchial recanalisation, one patient with extraction of bronchial tamponade due after bleeding and one unknown procedure; in addition, there was no intervention in one patient. Other interventions for S2 include two patients with removal of valves and one unknown procedure. Numbers in superscript indicate the number of unknown cases, numbers without superscript indicate data were available from the entire study population.

**Table A3:** Adverse events (AEs) during and after the intervention with subspecification of the observed events.

Adverse Events *			
	S1 (with LAP)	S2 (without LAP)	*p*-Value
	*n* = 39 (100%)	*n* = 35 (100%)	
**AE during intervention—*n* (%)**			n.s.
No	39 (100)	33 (94.3)	
Yes	0 (0)	2 (5.7)	
Desaturation	0 (0)	2 (5.7)	
**AE post-interventional—*n* (%)**			
No	24 (70.6)	25 (83.3)	n.s.
Yes	10 (29.4)	5 (16.7)	
Heart rate > 100/min	2 (5.9)	0 (0)	
Dyspnea	4 (11.8)	2 (6.7)	
Bronchospasm	0 (0)	1 (3.3)	
Desaturation	0 (0)	1 (3.3)	
Hoarseness	1 (2.9)	0 (0)	
Shivering	2 (5.9)	0 (0)	
Headache	0 (0)	1 (3.3)	
Hearing impairment	1 (2.9)	0 (0)	

* Adverse events are presented as absolute number and percentage (%) in relation to the known cases. AEs were unknown in five patients in S1 and five patients in S2.
